# Do Observed Teaching Behaviors Relate to Students’ Engagement in Physical Education?

**DOI:** 10.3390/ijerph18052234

**Published:** 2021-02-24

**Authors:** Alba González-Peño, Evelia Franco, Javier Coterón

**Affiliations:** 1 Social Sciences Applied to Physical Activity, Sport and Leissure Department, Faculty of Physical Activity and Sports Sciences—INEF, Universidad Politécnica de Madrid, 28040 Madrid, Spain; alba.gonzalez.peno@alumnos.upm.es (A.G.-P.); j.coteron@upm.es (J.C.); 2 Departament of Education, Research and Evaluation Methods, Faculty of Social and Human Sciences, Universidad Pontificia Comillas, 28049 Madrid, Spain

**Keywords:** behavioral engagement, teaching behavior, self-determination theory, physical education

## Abstract

Teachers’ behaviors can affect students’ engagement in the Physical Education (PE) setting. According to self-determination theory, teachers can rely on either a need-supportive or a controlling teaching behavior, and these behaviors will differently affect students’ outcomes. The main objective of this research was to analyse how teaching behaviors and some contextual variables influence students’ engagement in PE classes. The present study adds to the existing literature through an observation-based design in which real-life examples of need-supportive and thwarting teaching behaviors, as well as students’ engagement behaviors, have been identified. Thirty-seven different PE lessons were coded for 5-min intervals to assess the occurrence of 36 teaching behaviors and five students’ behaviors. Stepwise regression revealed that both structure during activity and relatedness support could predict student engagement in a positive way. Surprisingly, cold teaching also emerged as a direct predictor in the last step of the analysis. On the other hand, controlling and structure before activity behaviors inversely predicted students’ engagement. These four variables explained 39% of the variance in student engagement, whereas autonomy support did not correlate with student engagement. These new findings in the field not only confirm the known relevance of teaching behavior for students’ outcomes but also suggest an unexpected lack of influence of autonomy support on students’ engagement as well as an association between cold teaching and students’ engagement. Results are discussed in the light of new approaches, and some practical implications are provided.

## 1. Introduction

The physical education (PE) setting can be an ideal context to encourage the acquisition of healthy lifestyles and adherence to physical activity (PA) and sport throughout a pupil’s life development [1,2].

Student engagement is a multifaceted concept that reflects behavioral, emotional, and cognitive aspects [3]. When students are engaged in class, they listen, strive, and persist in the task, answer the questions the teacher asks or enjoy doing the proposed activities [4,5]. In contrast, when students are not engaged in the class, they do not display effort or persistence, they give up easily, they do not listen to the teacher, and they get bored [6]. Behavioral engagement has emerged as an important construct in the prediction of students’ performance and learning achievement [7]. Different authors have studied this topic, pointing out a positive consequence of the way in which the teacher interacts with his or her students [8,9].

The great interest of both researchers and educators in the understanding of factors that affect student engagement might be explained by the association found between this outcome and other physical activity related variables such as adherence and future intention to practice PA [10]. There is evidence that engagement will be affected by teacher–student interactions [11], and several studies have approached this relationship under the lens of self-determination theory (SDT).

SDT [12,13] offers an approach to explain how motivational processes affect several behavioural outcomes. It establishes a continuum of regulations that vary according to the degree of self-determination present in each of them and that goes from intrinsic motivation to amotivation. To promote the interiorization of these behaviors, the support of three basic psychological needs (BPN)—autonomy, competence, and relatedness—is crucial. The satisfaction of these BPN will determine the degree and type of motivation presented by each person. In this way, the increase in the perception of BPN satisfaction is associated with self-determined motivation, while frustration is related to extrinsic motivation and amotivation [13]. Recent studies conducted in PE and sport contexts have pointed out that students’ outcomes (e.g., need satisfaction, autonomous motivation) were positively influenced by a teacher’s autonomy support profile [14,15]. As a result, teachers’ autonomy support seems to be crucial in promoting students’ positive behaviors [16]. In this line, different authors, highlighting SDT postulates, have shown positive correlations between autonomous motivation and adaptive outcomes. Results have suggested that teachers influence students’ experiences in autonomy and competence needs, and both peers and teachers are associated with relatedness needs [17].

The students’ BPN satisfaction is thus affected by the students’ experiences in class, and teaching behavior seems to have an influence on it as well as on self-determined motivation [18] and greater satisfaction of BPN [19]. In line with SDT postulates, and according to previous works, teachers can foster students’ autonomy, competence, and relatedness through different need-supportive behaviors. [20,21,22]. Teachers who support autonomy seek to understand and respond to the perspective of students by offering opportunities to choose, work autonomously or perform tasks that meet their interests [23,24]. Competence can be fostered through the provision of structure. Previous studies have suggested that student engagement is affected by the structure provided by teachers [25]. Based on a description of structure as a multifaceted concept [26], Haerens et al. identified two facets depending on the specific moment it was provided in the lesson: structure before and structure during the activity [27]. Supporting structure before the activity involves, for instance, setting clear objectives, while structure during the activity considers providing effective feedback and information to support engagement (see Table S1). Lastly, the need of relatedness is nurtured by creating warm contexts where teachers are empathetic, caring, and understanding of their students [27,28,29]. On the other hand, by exerting thwarting behaviors, teachers can undermine students’ BPN [9,25,27]. When displaying need-thwarting behaviors, teachers exercise power as an authority by demanding respect, ignoring students’ perspectives and interests, or by pressuring them by referring to their self-confidence [30,31,32]. By being controlling or authoritarian, teachers can thwart student’s needs for autonomy, competence, and relatedness. By creating a cold environment where teachers act annoyed or unfriendly, needs for relatedness can be thwarted. In a chaotic environment, teachers are permissive and provide a few rules for adequate behavior, thwarting students’ needs for competence.

Different studies have shown the impact of teacher’s actions on students’ attitudes and behaviors through the analysis of students’ perception of teacher’s behaviour [7,18]. Student behaviors have been mainly addressed from two approaches. While some studies have relied on ecological measurements (e.g., accelerometry) [33], most of the existing research has addressed the relationships established between these variables through self-reported measures in which researchers do not interfere with participants’ responses [34,35]. Although these assessment tools are useful when they present adequate values of reliability and validity given their low economic cost and ease of administration, there is evidence that the data collected could be biased by the need for social approval, conceptualized as social desirability [36]. The analysis of behaviors through observation is positioned as an interesting alternative that allows us to deepen the knowledge of the interactions that occur in real teaching situations, avoiding the bias mentioned above.

In this way, studies that use observational methodology in the field of PE, PA, and sport are characterised by perceptiveness and spontaneity of the observed behavior—which allows a more in-depth approach to the reality analysed, the habituality in the context, and the development of customised observation instruments [37]. However, as mentioned earlier, the literature is still scarce in studies using observational methodology for the analysis of teacher–student interactions in the classroom, opting for the use of questionnaires which ask about the perception of the variables analysed.

Therefore, the main objective of this study was to analyse how teaching behaviors influence students’ engagement during PE classes. The specific contribution of this study to the existing literature concerning teacher–student interactions is based on (a) the use of observational methodology for both teachers’ and students’ variables and (b) the consideration of contextual variables as potential predictors of students’ engagement.

## 2. Materials and Methods

The present study constitutes a descriptive, post facto investigation [38]. In order to obtain more precise data and given their high degree of flexibility, the observational methodology was based on indirect systematic observation [39,40] to analyse the teaching behavior and the behavioral engagement of students. Contextual variables affecting each class were both observed and confirmed by asking the PE teacher.

The nature and requirements of the study justified the use of a Nomothetic, Monitoring, and Multidimensional (N/M/M) type design [37,39]. The design was (a) nomothetic because the variables of seven PE teachers were analysed individually; (b) monitoring because data collection took place in successive sessions (intersessionally), recording the entire class without interruption (intrasessionally)—the modifications and stability of these being of interest; and (c) multidimensional because it presents a behavioral flow with several levels of response.

### 2.1. Participants

The sample of this study was made up of male (*n* = 4) and female (*n* = 3) PE teachers, with an average of 12.22 years of experience (SD = 8.25), belonging to public, private, and subsidised centres in Spain and Argentina, whose age ranged from 27 to 55 years (M = 36.86; SD = 9.26) (see Table 1). One group class taught by each of these teachers also participated in the study. The participant groups were composed on average by 19.16 ± 4.94 students, aged from 12 to 16 years. Finally, 709 students were included.

Schools that took part in the research were located in Buenos Aires and Madrid regions. All of them presented low socioeconomic levels. In both countries, PE is a compulsory subject in the curriculum, and at least two hours per week are required according to the class schedule. In Spain, mixed education is the most common situation due to current education laws; segregated education is frequently seen in Argentina’s schools. The sample of the present study was composed of both mixed and segregated groups.

### 2.2. Procedure

Data were collected from non-probability samples of teachers and students in compulsory secondary education after receiving approval from the University Ethics Committee. All participants were in agreement with the ethical guidelines of the American Psychological Association [41]. The management teams and heads of department of the different participating schools were contacted and informed about the study’s objectives and their collaboration was requested. Having identified the centres willing to participate in the study, teachers, students, and students’ parents or legal guardians were also informed about the study. Once the informed consent was obtained from the participants and the students’ parents or legal guardians, data were collected.

The collection of observational data was carried out during 37 sessions, obtaining a total of 358 codifications regarding teaching behavior, and engagement. For the video and audio recording, an SJCAM 5000+ video camera and an audio recorder smartphone were used with a microphone placed on the teachers’ clothing so as not to interfere with their work. Adobe Premiere Pro software was used to digitalize the records, which showed the sessions from the beginning to end by means of a continuous recording from the moment the teacher started in the practice scenario until the end of the session. Subsequently, teaching behaviors and students’ engagement were coded with LINCE PLUS [42] qualitative data analysis software, and the following steps were taken:

1. Design of the final observation sheet to allow for teaching behavior coding and student engagement.
2. Coding of teaching behaviors in 5-min intervals in each session.
3. Coding of the student’s engagement in 5-min intervals in each session.
4. Export of results and data analysis.

### 2.3. Instruments

#### 2.3.1. Teaching Behavior

Teaching behavior was assessed using the scale used by Haerens et al. [27] and Van den Berghe et al. [32]. An external evaluator, who was trained in this process, coded 5-min intervals from the beginning to the end of each session. The scale includes a description for need-supportive teaching behavior that reflects four dimensions with 20 items: three items for autonomy support (α = 0.83) (e.g., “Offer choice to all students”), five items for relatedness support (α = 0.83) (e.g., “Teacher puts effort and energy into the lesson”), five items for structure before the activity support (α = 0.64) (e.g., “The teacher provides variation in exercises or within exercises”), and seven items for structure during the activity support (α = 0.48) (e.g., “The teacher offers the students (apart from instruction) new guidelines, tips, and advice during the exercises”). Need-thwarting teaching behavior reflected three dimensions with 16 items: seven items for the controlling dimension (α = 0.46) (e.g., “Teacher is irritated, loses his patience”), five items for the cold dimension (α = 0.68) (e.g., “Teacher is acting unfriendly and cold”), and four items for the chaotic dimension (α = 0.58) (e.g., “Teacher allows chaos, and leaves the students to it”). Each item was coded on a four-point scale from 0 (never observed) to 1 (sometimes observed), 2 (frequently observed), and 3 (observed all the time).

#### 2.3.2. Students’ Behavioral Engagement

Students’ behavioral engagement was assessed using the same scale used by Aelterman et al. [10] and proposed by Reeve et al. [5]. This variable was evaluated by means of observation of the whole class by an external evaluator recoding the 5-min intervals from the beginning to the end of each session. Student engagement (α = 0.79) included five items: listening to the teacher, effort, asking questions, persistence, and enjoyment. A four-point scale from 0 (never) to 3 (always) was used.

In total, 358 intervals were coded, and the coding process took approximately 180 min per class, for a total of 111 h of coding. To assess the interobserver reliability of the observed items of teaching behavior and student engagement, two previously independently trained observers coded 20 identical intervals of the PE classes. To assess intra-observer reliability, one observer twice coded 15 intervals with a 2-week gap between each analysis. The observers were familiar with SDT and research in the area of PE. Intra-observer and interobserver reliability were calculated using intraclass correlation coefficients (ICC). Van den Berghe et al. [32] showed good intra (0.95) and interobserver reliability (0.87), as well as internal consistency (0.80) for need- and thwarting-support teaching behaviors. Aelterman et al. [10] also illustrated good intra- and interobserver reliability for the scale that was used to codify student engagement. In the present study, inter-rater reliabilities of all retained factors ranged from 0.81 to 0.85; the intra-rater reliabilities values ranged from 0.79 to 0.86.

### 2.4. Data Analysis

Descriptive analyses and bivariate correlations of all variables studied were performed. To analyze the relationship between teaching behavior and behavioral engagement, a stepwise regression was performed using behavioral engagement as the dependent variable. Stepwise regression is a method of fitting regression models in which the choice of predictive variables is carried out by an automatic procedure [43].

More specifically, a forward selection approach was selected. This technique involves starting with no variables in the model and testing the addition of each independent variable using a chosen model fit criterion. In this case, the criterion was a *t*-test and adjusted R^2^. Thus, variables are included in the model as long as they provide significant improvement of the fit, starting with those whose inclusion gives the most statistically significant improvement, and repeating this process until no additional variable improves the model to a statistically significant extent.

The data were processed using the SPSS 20.0 statistical package.

## 3. Results

Table 2 presents the descriptive statistics for the study variables. In general, the scores were high for student engagement. Teaching behaviors that promote structure before and during the activity were also high. Pearson correlation analyses were conducted to study the relationship between the variables under study. Engagement positively correlated with structure and relatedness support behaviors but negatively with controlling behavior. A positive correlation was also found for structure before the activity support with relatedness support behavior. Likewise, the structure during the activity-support behavior was positively correlated with the relatedness behavior. These two last behaviors were negatively correlated with the controlling behavior. Relatedness-support behavior was positively correlated with the structure before the activity and structure during the activity support behaviors, but negatively correlated with the controlling behavior. Cold teaching correlated positively with controlling support and negatively with the structure before the activity and relatedness support. Finally, chaotic teaching correlated positively with autonomy support and cold teaching, but negatively with the structure during the activity and controlling support behaviors.

Table 3 shows the results of the stepwise regression analysis testing the prediction of teaching behavior for students’ engagement. The relatedness support behavior emerged as the strongest positive predictor (β = 0.70, *p* < 0.001), explaining 30% of the variance. In the second step, the model using relatedness support (β = 0.53, *p* < 0.001) and structure in the activity (β = 0.40, *p* < 0.001) predicted 36% of the explained variance of engagement. Step 3 included, in addition to relatedness (β = 0.55, *p* < 0.001) and structure in the activity (β = 0.43, *p* < 0.001), structure before the activity as a negative predictor (β = −0.12, *p* < 0.05). Step 4 included relatedness support (β = 0.52, *p* <0.001) and structure during the activity (β = 0.44, *p* < 0.001) as positive predictors, compared with structure before the activity (β = −0.14, *p* < 0.01) and controlling teaching (β = −0.36, *p* < 0.05) as negative predictors. It accounted for the 38% of the explained variance. The resulting (step 5) model reflected that both relatedness support (β = 0.53, *p* < 0.001) and structure during the activity (β = 0.43, *p* < 0.001) would positively predict the students’ engagement, and the structure before the activity (β = −0.13, *p* < 0.05) and controlling teaching behaviour (β = −0.39, *p* < 0.01) would negatively predict the aforementioned dependent variable. This last step added cold teaching (β = 0.65, *p* < 0.05) as a positive predictor of the students’ engagement and accounted for the 39% of the explained variance.

## 4. Discussion

The aim of this study was to analyse the predictive role of teaching behaviors (autonomy-, structure before the activity-, structure during the activity-, relatedness-, and controlling-support) in PE class.

One of the main contributions of the present work is the use of observational methodology instead of self-reported questionnaires, which allows a more in-depth approach to the reality under analysis (in this case, the PE setting). Overall, the findings suggested that the engagement students display in PE classes is positively associated with need-supportive behaviors and is negatively related to controlling behavior. This is in line with previous findings. Several studies in recent years have shown the influence of teaching behaviors on behavioral aspects through BPN improvement, which, in turn, improves motivation [21,44]. Thus, when a teacher uses a motivating teaching behavior, students perceive greater support for autonomy and less control. Therefore, the satisfaction of their psychological needs and their engagement improve, and their levels of de-motivation decrease.

Firstly, and as expected, this study showed that structure during activity support and the relatedness-support behaviors showed a positive relationship with behavioral engagement. When teachers support the structure during the activity, they are expected to provide adequate information, clarify their expectations, and offer clear guidelines before the activities, offering help during the proposed activity or providing positive feedback after the successful completion of the activity. The relatedness-support behavior is characterised by those qualitative and quantitative aspects related to the student–teacher interaction, referring to the degree of teacher involvement or the way he/she communicates with his/her students [32]. In this line, other studies have suggested that certain strategies may favour the use of behaviors that support the structure during the activity and the relatedness. The structure during the activity support is nurtured, for example, by the use of appropriate feedback related to the task objective with a positive value. As some authors have indicated [45,46], feedback is a key aspect for a student’s motivation and, consequently, for his/her engagement. Brehaut et al. [47] suggested different strategies for teachers to implement to provide multiple instances of feedback, as soon as possible and at a frequency informed by the number of new students’ task attempts, providing individual rather than general data or choosing comparators that reinforce desired behavior change. They also recommended preventing defensive reactions to feedback including positive messaging along with negative, or encourage self-assessment around target behaviors before receiving feedback, which will allow constructing feedback through social interactions. The buddy scheme technique based on the interaction of students with different competence levels addressing a certain task can nurture relatedness need satisfaction and, therefore, their engagement during the classes, as suggested by several authors [48,49,50]. Finally, using students as positive role models during an assignment, showing enthusiasm and paying attention to what students say are techniques that can be used by the teacher to support the structure during the activity and relatedness [32].

On the other hand, although this relationship was hypothesized, teaching autonomy-supportive behavior was not related to engagement. These results are not in line with those suggested by other works which point out the benefits of autonomy support for motivation [51], perseverance [52], and well-being [53]. Recently, a circumplex approach was proposed that has advanced the understanding of teaching behaviors [54,55]. This model interprets a two-dimensional structure. The first one is located on the X axis and can be interpreted as need thwarting versus need support, with control and chaos yielding negative coordinates and autonomy support and structure yielding positive coordinates in this dimension. The second dimension is located on the Y axis and can be interpreted as the level of teacher directiveness, with autonomy support and chaos items having negative coordinates and control and structure items having positive coordinates in this dimension. Each item is composed of two approaches or teaching behaviors: autonomy support (participative or attuning), structure (guiding or clarifying), controlling (demanding or domineering), and chaos (abandoning or awaiting). It was found that each approach correlated more strongly with its adjacent one. In the model, the dimension of participative (typical of autonomy-supportive teachers) borders on awaiting (characteristic of chaotic teachers); thus, a similarity between these two approaches could be assumed. Therefore, a teacher who supports autonomy could be perceived as a chaotic one. In this sense, the observation tool proposed by Van den Berghe et al. [32] presents some items of the autonomy dimension that could be similar to the characteristics of an awaiting teacher. For example, “offering the opportunity to face problems, to practice autonomously, to experiment, to work and solve problems on their own without interference from the teacher” could mean that students perceive autonomy as a chaotic environment where the teacher has no control over what happens in the classroom, as is characteristic of an awaiting teacher. This ambiguity regarding these two dimensions could explain the lack of a relationship between autonomy and engagement. Our findings suggest that to encourage an attuning approach rather than a participative one would be more beneficial to support students’ autonomy. From this participative approach, an environment is created where students’ interests are nurtured by trying to find activities that are more interesting to them, accepting and understanding their perspective, and providing informed explanations that are meaningful to students [55]. It has been suggested that different conditions may encourage a teacher to develop autonomy-supportive behaviors. In line with various authors, a well-structured and warm work environment where the teachers can take the initiative, are allowed to express their ideas and opinions, know what is expected from them, or are informed by the management of those good or effective practices that have been carried out during their performance in class, will also favour the use of more motivating behaviors supporting autonomy [56]. It would be interesting for future studies to address how different backgrounds are related to teaching behaviors established in a circumplex approach to gain further understanding of the implications of this more fine-grained approach. Furthermore, related to autonomy support, cold teaching also emerged as a direct predictor in the last step of the analysis.

The results of the present research showed a negative relationship between the structure before the activity support behavior and students’ engagement. Again, the circumplex approach may give us some clues to better understand the lack of an expected association. If we take a look at this model, we can observe that the clarifying approach (belonging to structure) is adjacent to the control-demanding approach [54]. In this way, various authors suggested that when the teacher uses structure support behaviors, he or she will be more directive, leading to interactions with the student. This fact could be interpreted as a behavior that, in an attempt to support the needs, demands a disciplinary approach and presents learning expectations that could frustrate them, establishing interesting similarities between the clarifying approach of the structure support behavior and the demanding approach of the controlling behavior [55]. For example, the observation tool item [32] “gives clear verbal instructions” could be perceived as a demand for discipline, pointing out obligations and sanctions if students do not comply. Therefore, it is of great importance to emphasize these findings in order to provide teachers with the necessary tools to clearly communicate the objectives of the task to the student without inhibiting him/her in his/her practice, thus avoiding the demanding approach. Again, this ambiguity regarding these two dimensions could explain the negative relationship between support given to the structure before the activity and the engagement.

In line with the literature and the finding discussed above, the results showed an inverse relationship between controlling behavior and student engagement levels. Numerous studies in recent years have shown the relationship between a controlling behavior, in which BPN are likely to be thwarted, and a variety of disadaptive students’ outcomes, suggesting a dark side in teaching [18,32,35]. Therefore, when a teacher makes use of a controlling behavior and students perceive it as such, their BPN are frustrated, leading to controlled motivation, demotivation, and defiance, factors which are related to a lack of engagement in the classroom. Along the same lines, De Meyer et al. [18] studied the relationship between the controlling behavior and the motivation of students, finding that with those teachers who exhibit more controlling behavior, students feel more pressured during the PE classes, which is associated with demotivation. It seems that teacher’s motivation is a conditioning aspect of using controlling behavior [32]. Considering the relevance of teachers’-controlled motivation on the motivation of their students, several studies have tried to focus on the teacher’s background that leads to this motivational state. For example, Abós et al. [57] found that teachers who experience burnout syndrome tend to be more unmotivated, so they will put more pressure on and control their students. These aspects, such as pressure, control, and demand, are characteristic of controlling behaviors, and, as Van den Berghe et al. [56] noted, are associated with controlled motivation. In this line, more evidence would be needed such as that of Coterón et al. [58], who analysed different backgrounds and their influence, finding significant relationships between teacher NPB satisfaction and student engagement, suggesting that the impact of certain external pressures on teachers not only affects their way of teaching but also the behavior of their students.

Finally, the findings suggested a relationship between chaotic teaching and engagement. However, the results did not reflect meaningful changes in adjusted R^2^ values. In a deeper analysis of these codes, we found that some items seemed to be not specific enough and in some cases reflected general behaviors not directly connected with teacher intentionality but with issues outside the class (e.g., somebody came and asked the teacher about a request authorization). It would be interesting to keep working on the design of observation tools that allow researchers to obtain more reliable results in the PE context by improving some items that seems to differ with teacher intentionality.

This study represents a further step in the understanding of the interactions that take place between teachers and students and highlights the importance of caring for both teachers, because of the close relationship with their professional performance, and students, because of their influence on teaching performance. Most of the research aiming to test the effect of need-supportive interventions, despite having proposed strategies fostering the different needs, has measured the impact of these strategies as a whole, lacking information about what is the independent role of supporting each BPN in different students’ outcomes [59]. In this line, the findings of the present work suggest a differential role of supporting each BPN in an individual way. The results of the study have some interesting practical implications. Firstly, it would be helpful that PE teachers are aware of how relevant their practices are in shaping their students’ behaviors. In this line, the design and development of training programs including some thought-provoking evidence in this respect would be of great value. The presence of specific and feasible strategies to foster need-supportive behaviors in these programs could also contribute to the improvement of the teacher–student interactions’ quality from an SDT perspective.

It should be noted that the present investigation has some limitations. Although the alpha values in some dimensions were lower than the acceptable value (<0.70), we found it interesting to include those dimensions for the purpose of the research, taking as reference the works previously carried out by Van den Berghe et al. [60], who also obtained similar values. These same authors measured pupil-to-pupil engagement by choosing a different one for each session, while in this study, the whole class was measured in each session. It would be interesting to use this instrument again to know if measuring this variable individually or in a group provides different results. The sample was composed of participants from different countries and different types of education: mixed and segregated. While previous studies have suggested that no significant differences have been found in motivational profiles shown by students from Spain and Argentina [61], it would be interesting for future studies to analyse whether the nature of the education (mixed or segregated) could affect students’ outcomes in the PE setting. The role of teachers’ characteristics in students’ outcomes has also been examined in previous studies: teaching for longer might be associated with more burnout, which, in turn, will negatively affect students’ behaviors [62]. Furthermore, other studies have shown that students better value PE when the teacher is male [63]. Different studies have also suggested that the context can affect engagement as well as other psychological and behavioral outcomes of students, showing that students’ competence perceptions were influenced by the content [64]; therefore, PA levels were different depending on the class content [65]. However, the sample size is not large enough to draw any conclusion about associations between contextual variables and students’ engagement. It is finally worth mentioning that the sample was mostly composed of teachers adopting need-supporting behaviors. It would be interesting to explore larger and more heterogeneous samples to further understand the teacher-student interactions.

However, this study allows us to understand the extent of the effect that different contextual variables have on student performance, which may be of interest to both researchers and teaching staff. As a future research study, it would be interesting to deepen observational methodology procedures in PE classes to contrast the real setting with participants’ perceptions. Furthermore, future studies could rely on observations of variables referring to both teachers and students such as adaptive outcomes (e.g., motivation, need satisfaction) in order to find out how students’ and teachers’ behaviors influence each other in the teaching and learning processes during PE classes.

## 5. Conclusions

This study explored the relationship between student engagement, teaching behavior, and contextual variables. The results obtained in the present study indicate as a general trend that need-support teaching behavior has a positive influence on students’ behavioral engagement. This investigation thus contributes to the understanding of possible behavioral patterns of both teachers and students, which could guide the design and implementation of strategies within the PE setting.

## Figures and Tables

**Table 1:** Contextual variables.

T	N	G	A	YE	MG	SG	TS	IS	5-Min	T
T01	Spanish	Male	55	30	5	0	4	2	47	5
T02	Spanish	Male	41	15	6	0	2	4	53	6
T03	Spanish	Female	30	4	5	0	2	4	48	5
T04	Argentine	Male	32	10	0	5	3	2	51	5
T05	Argentine	Female	37	10	2	3	3	2	50	5
T06	Argentine	Female	27	5	2	4	4	2	57	6
T07	Argentine	Male	36	11	2	3	3	2	52	5

Note: T = Teacher; N = Nationality; G = Gender; A = Age; YE = Years of Experience; MG = Mixed Group Sessions; SG = Segregated Group Sessions; TS = Team Sports Sessions; IS = Individual Sports Sessions; 5-Min = Number of 5-min intervals; T = Total Sessions.

**Table 2:** Descriptive statistics and correlations between the analysed teacher and student variables.

Variables	M	DE	1	2	3	4	5	6	7	8
1. Engagement	1.92	0.65	---	0.13 *	−0.10	0.47 **	0.55 **	−0.21 **	0.03	0.01
2. Autonomy support	0.77	1.04		---	0.46 **	0.02	0.23 **	−0.39 **	−0.04	0.22 **
3. Structure before the activity support	0.83	0.62			---	0.26 **	0.27 **	−0.20 **	−0.16 **	0.09
4. Structure during the activity support	1.06	0.46				---	0.46 **	−0.06	−0.04	−0.11 *
5. Relatedness support	2.71	0.50					---	−0.25 **	−0.12 *	−0.02
6. Controlling support	0.23	0.21						---	0.16 **	−0.13 *
7. Cold teaching	0.04	0.01							---	0.12 *
8. Chaotic teaching	0.12	0.23								---

Note: ** The correlation is significant at the 0.01 level (bilateral). * Correlation is significant at the 0.05 level (bilateral).

**Table 3:** Stepwise regression analysis for students’ behavioral engagement.

Variables	b	SE	Adjusted R^2^	t	*p*
Step 1			0.30		
Relatedness support	0.55	0.06		11.97	0.000
Step 2			0.36		
Relatedness support	0.42	0.06		8.45	0.000
Structure during the activity support	0.28	0.07		5.72	0.000
Step 3			0.37		
Relatedness support	0.44	0.06		8.78	0.000
Structure during the activity support	0.30	0.07		6.06	0.000
Structure before the activity support	−0.11	0.05		−2.40	0.017
Step 4			0.38		
Relatedness support	0.41	0.06		8.08	0.000
Structure during the activity support	0.31	0.07		6.29	0.000
Structure before the activity support	−0.13	0.05		−2.77	0.006
Controlling teaching	−0.12	0.14		−2.54	0.011
Step 5			0.39		
Relatedness support	0.42	0.06		8.26	0.000
Structure during the activity support	0.31	0.07		6.24	0.000
Structure before the activity support	−0.12	0.05		−2.52	0.012
Controlling teaching	−0.13	0.14		−2.81	0.005
Cold teaching	0.10	0.29		2.26	0.024

Note: b = standardised beta coefficients; SE = standard errors.

## Data Availability

Data available on request due to restrictions eg privacy or ethical.

## Supplementary Materials

The following are available online at https://www.mdpi.com/1660-4601/18/5/2234/s1, Table S1: Teaching Behaviors and students’ engagement observation tool.
