# Supplementary material for: Do Observed Teaching Behaviors Relate to Students’ Engagement in Physical Education?
Source: Int J Environ Res Public Health. 2021 Feb 24;18(5):2234. doi: 10.3390/ijerph18052234 (PMC7967671; doi:10.3390/ijerph18052234)
Supplement: Supplementary file 1 [file ijerph-18-02234-s001.pdf]

Supplementary

Table S1. Teaching Behaviors and students' engagement observation tool.

Items	Variables
1. The students pay attention during this PE class	Engagement
2. The students put effort in the activities and exercises	Engagement
3. The students ask questions about the exercises	Engagement
4. The students don't give up easily during challenging tasks	Engagement
5. The students seem to enjoy this PE class	Engagement
6. The teacher offers choice to all students	Autonomy support
7. The teacher gives students the opportunity to practice independently and to solve problems on their own, without interfering	Autonomy support
8. The teacher applies differentiation	Autonomy support
9. The teacher offers the students a rationale for tasks and exercises	Structure before the activity
10. The teacher gives an overview of the content and structure of the lesson	Structure before the activity
11. The teacher gives clear (verbal) instructions	Structure before the activity
12. The teacher provides variation in exercises or within exercises	Structure before the activity
13. The teacher demonstrates the tasks himself, is a 'model' for the students	Structure before the activity
14. The teacher offers the students a rationale for tasks and exercises	Structure during the activity
15. The teacher monitors if the students consequently live up to the (verbal) instructions	Structure during the activity
16. The teacher offers students (apart from instruction) new guidelines, tips and advice during the exercises	Structure during the activity
17. The teacher provides positive feedback	Structure during the activity
18. The teacher uses students as positive role models	Structure during the activity
19. The teacher offers help during exercises	Structure during the activity
20. The teacher addresses students by their first name when the opportunity occurs	Structure during the activity
21. The teacher is physically nearby the students	Relatedness support
22. The teacher is enthusiastic and eager	Relatedness support
23. The teacher puts effort and energy into the lesson	Relatedness support
24. The teacher takes the perspective of students into account, is empathic	Relatedness support
25. The teacher pays attention to what the students are saying	Relatedness support
26. The teacher exercises power over the students by interfering and demanding respect	Controlling
27. The teacher commands students, uses controlling language and imperatives	Controlling
28. The teacher is irritated, loses his patience	Controlling
29. The teacher yells at the students	Controlling

30. The teacher pressures the students by making an appeal to their self-confidence or pride or induces feelings of guilt and shame	Controlling
31. The teacher uses destructive criticism when students not acting in the way the teacher expects them to	Controlling
32. The teacher does not allow input from the students or reacts negatively to their input	Controlling
33. The teacher does not pay much attention to the students	Cold
34. The teacher is acting unfriendly and cold	Cold
35. The teacher takes distance from the students, is detached	Cold
36. The teacher is distracted by activities not related to the students or the content of the lesson	Cold
37. The teacher is acting inconvenient and annoying towards students	Cold
38. The teacher loses time with the reorganization of groups, equipment…	Chaotic
39. The teacher doesn’t know the students’ names	Chaotic
40. The teacher allows chaos, and leaves the students to it	Chaotic
41. The teacher uses an illogical and inconsistent structure during the warming up and activity or in the transitions between exercises	Chaotic
